# Effectiveness of dry needling and high-volume image-guided injection in the management of chronic mid-portion Achilles tendinopathy in adult population: a literature review

**DOI:** 10.1007/s00590-017-1957-1

**Published:** 2017-04-19

**Authors:** F. A. Chaudhry

**Affiliations:** 1 Department of Trauma and Orthopaedics, Russells Hall Hospital, Dudley, DY1 2HQ UK; 2 The University of Warwick, Coventry, CV4 7AL UK

**Keywords:** Achilles tendinopathy, High-volume image-guided injection, Dry needling

## Abstract

Achilles tendinopathy is a common overuse condition affecting the adult population. The incidence is on the rise because of greater participation of people in recreational or competitive sporting activities. There are several treatment options available both non-operative and operative. Ultrasound-guided dry needling and high-volume image-guided injection is relatively a new procedure. The aim of this study was to find out the effectiveness of dry needling and HVIGI in the management of mid-portion chronic Achilles tendinopathy by performing a literature review. Search strategy was devised to find the suitable articles for critical appraisal using the electronic databases. Four articles were selected for critical appraisal, and these papers showed good short- to long-term results of image-guided high-volume injection in the management of Achilles tendinopathy. We conclude that high-volume image-guided injection is effective in the management of Achilles tendinopathy. It provides good short- and medium-term relief of symptoms. It should be considered as one of the many options available for this condition.

## Introduction

 Tendinopathies are soft tissue disorders related to tendons [1]. Non-insertional Achilles tendinopathy occurs because of failure to mediate the repair and degeneration processes, leading to pain and disability [2]. The use of injectable substances such as platelet-rich plasma, autologous blood, polidocanol, corticosteroids and aprotinin in and around tendons is popular for Achilles tendinopathy, but evidence to support their role is minimal [3]. Ultrasound-guided high-volume image-guided injection (HVIGI) is relatively a new technique, and the literature on this technique is limited. This technique can be combined with dry needling.

### Background

Achilles tendinopathy is a common overuse condition affecting the adult population. The incidence is on the rise because of greater participation of people in recreational or competitive sports [2]. The general population has an incidence of 2.35 per 1000 people, which is roughly equivalent to more than 150,000 people in the United Kingdom every year [4]. It is a clinical triad of pain, impaired performance and swelling [5]. The main stimulus to cause tendinopathy is excessive repetitive overload; however, about one-third of the studied group did not participate in the vigorous physical activities [6]. During running, forces up to 12.5 times body weight passes through the Achilles tendon which may contribute to tendinopathy [7].

### Aetiology

Tendinopathies have been linked to poor vascularity, poor flexibility, genetic make-up, endocrine and metabolic factors [2]. Use of quinolone antibiotic has also been linked [8]. The pathological stimulus is excessive loading of the tendon during physical activity. Various intrinsic factors include poor vascularity, dysfunction of the gastrocnemius-soleus, age, gender, body weight, pes cavus and lateral instability of the ankle [2]. Changes in training pattern, poor technique, previous injuries, footwear and environmental factors such as training on hard, slippery or slanting surfaces are extrinsic factors [2]. The term “tendinopathy” is a description of a clinical condition which includes both pain and pathological process associated with overuse in and around the tendon. “Tendinosis” and “tendonitis” are histopathological descriptive terms and should ideally be used after the histopathological confirmation [9]. The pathological process of Achilles tendinopathy is considered to be a degenerative rather than inflammatory [10].

It is categorised into two types, insertional or non-insertional. Insertional type is less common and affects 20–25% of cases [11]. The predisposing factors for insertional type include increasing age, inflammatory arthropathies, corticosteroid use, diabetes, hypertension, obesity, gout, hyperostotic conditions, lipidaemias and quinolone antibiotics [12]. Symptoms are more proximal in the non-insertional type [13]. It accounts for 70–75% cases presenting, and it affects 9% of recreational runners. It can end the careers of 5% of professional runners [5].

### Treatment options

There are many treatment options (Table 1). A recent meta-analysis has advocated that eccentric loading exercises are the gold standard in the management of Achilles tendinopathy [14] although other studies have pointed that there are not enough good-quality randomised controlled trials available in the literature on this subject [3, 12].

**Table 1:** Treatment options

Non-operative options	Operative options
*Physical therapies*	*Tenotomies*
Eccentric exercises	Multiple percutaneous longitudinal tenotomies
Shock wave therapy	Radiofrequency microtenotomy
	Neovessel destruction
*Injectable substances*	*Arthroscopic*
High-volume injections	Arthroscopic debridement
Corticosteroids	*Surgery*
Local anaesthetic agents	Tendon debridement
Platelet-rich plasma	Tendon decompression
Autologous blood injection	Tendon transfer
LMW heparin	Gastrocnemius lengthening
Sclerotherapy	
*Other*	
GTN Patches	

Source [3, 12]

### What is dry needling and high-volume image-guided injection (HVIGI)

Dry needling involves repeated needling in the abnormal tendon to promote an inflammatory response. Repeated passage of the needle produces physical trauma to the tendon which in turn causes internal haemorrhage leading to an inflammatory response which causes the formation of granulation tissue. This granulation tissue strengthens the tendon [15]. The local anaesthetic is injected around the area before performing the dry needling. There have been early reports of good results of ultrasound-guided needle tenotomy with corticosteroid injection for the treatment of common extensor tendons [16]. Jeffrey et al. evaluated the safety and short-term effectiveness of ultrasound-guided needle tenotomy in other tendons without the use of corticosteroids, because of the fear of tendon rupture [17]. Their study included 14 tendons, but only 4 of them were Achilles tendons.

High-volume image-guided injection (HVIGI) consists of normal saline, local anaesthetic and corticosteroids. It tends to improve amount of pain and improve function in patients suffering with Achilles tendinopathy [20]. The procedure is described as using an aseptic technique, a 21-gauge needle is inserted under real-time ultrasound guidance between the anterior aspect of the Achilles tendon and Kager’s fat pad, targeting the area of maximal neovascularisation. Then a mixture of 10 mL 0.5% bupivacaine hydrochloride and 25 mg of hydrocortisone acetate is injected, followed by 4 × 10 mL of injectable normal saline [20–22]. Some authors have described using more volume and without adding corticosteroids [23].

### Other closely related procedures

There are few closely related procedures that are used for this condition. Sclerotherapy is a procedure that involves injecting a chemical into a blood vessel. The theory behind its use is to sclerose the vessels and eradicate the pain generating nerve fibres [2]. Autologous blood injection involves reinjection of patients own blood. The proposed mechanism of action of this is that the cytokines and growth factors within the injected blood help to stimulate tissue healing and production of type 1 collagen, promoting tendon repair [18].

### Aim

The aim of this literature review was to find out the evidence for the use of high-volume injections in the treatment of chronic mid-substance Achilles tendinopathy.

### Research question

The research question to do the literature review was produced using the PICO model (Table 2).

**Table 2:** PICO model

Population	Intervention	Comparison	Outcome
Adult population with	Dry needling	Other minimally invasive treatment methods	*Primary outcome*
chronic Achilles tendinopathy			
	High-volume injection		Pain score
			*Secondary outcome*
			Functional outcome measure complications

## Materials and methods

A search strategy was devised to answer the research question. The PRISMA guidelines were used to conduct the review [19].

The primary objective was to review the effectiveness of high-volume image-guided injection in the management of Achilles tendinopathy. Therefore, the primary outcome measure was pain scores such as visual analogue score (VAS) and the secondary outcome was functional score such as Victorian institute of sports assessment for Achilles tendon (VISA-A).

### Information sources

The articles were identified using the electronic databases of Embase (1980–March 2017), Medline (1950–May 2017) and PubMed. The Ovid search engine was used. The search was carried out on 19 March 2017. An additional search was carried out to find out any unpublished literature using the register of current controlled trials database for recently completed trials (http://controlled-trials.com/isrctn). One study was found, titled the “High Volume Saline Injections for Achilles Tendinopathy (ISRCTN87144429 DOI 10.1186/ISRCTN87144429)” but it has not been published yet. A hand search was also undertaken using the reference lists of review papers that were evaluated to identify any additional relevant articles.

### Keywords

The keywords used were Achilles tendinopathy, Achilles tendonitis, high-volume image-guided injection and dry needling. Two searches were performed (Tables 3, 4). The terms were used under mesh headings. “AND” and “OR” terms were used to combine the keywords.

**Table 3:** Search 1 (19.03.2017)

	Searches	Results	Type
1	Achilles tendinopathy.mp. or exp Achilles tendinitis/	1486	Advanced
2	Exp Achilles tendinitis/Achilles tendinitis.mp.	1390	Advanced
3	1 or 2	1612	Advanced
4	Dry needling.mp.	408	Advanced
5	3 and 4	11	Advanced

**Table 4:** Search 2 (19.03.2017)

	Searches	Results	Type
1	Achilles tendinopathy.mp. or exp Achilles tendinitis	1486	Advanced
2	Exp Achilles tendinitis/Achilles tendinitis.mp.	1390	Advanced
3	1 or 2	1612	Advanced
4	High-volume image-guided injections.mp.	3	Advanced
5	3 and 4	3	Advanced

### Inclusion and exclusion

Studies selected for review were original articles. The inclusion criteria were:

1. Ultrasound-guided dry needling and high-volume injection studies
2. At least 10 patients were included in the study
3. Adult population age more than 18 years
4. The articles were published in English language
5. The full text was available for review

The exclusion criteria were:

1. Systematic reviews or meta-analysis
2. Animal or experimental studies
3. Conference abstracts
4. Studies using other non-operative methods such as eccentric loading exercises, sclerotherapy and prolotherapy.

### Article selection

**Fig. 1:**
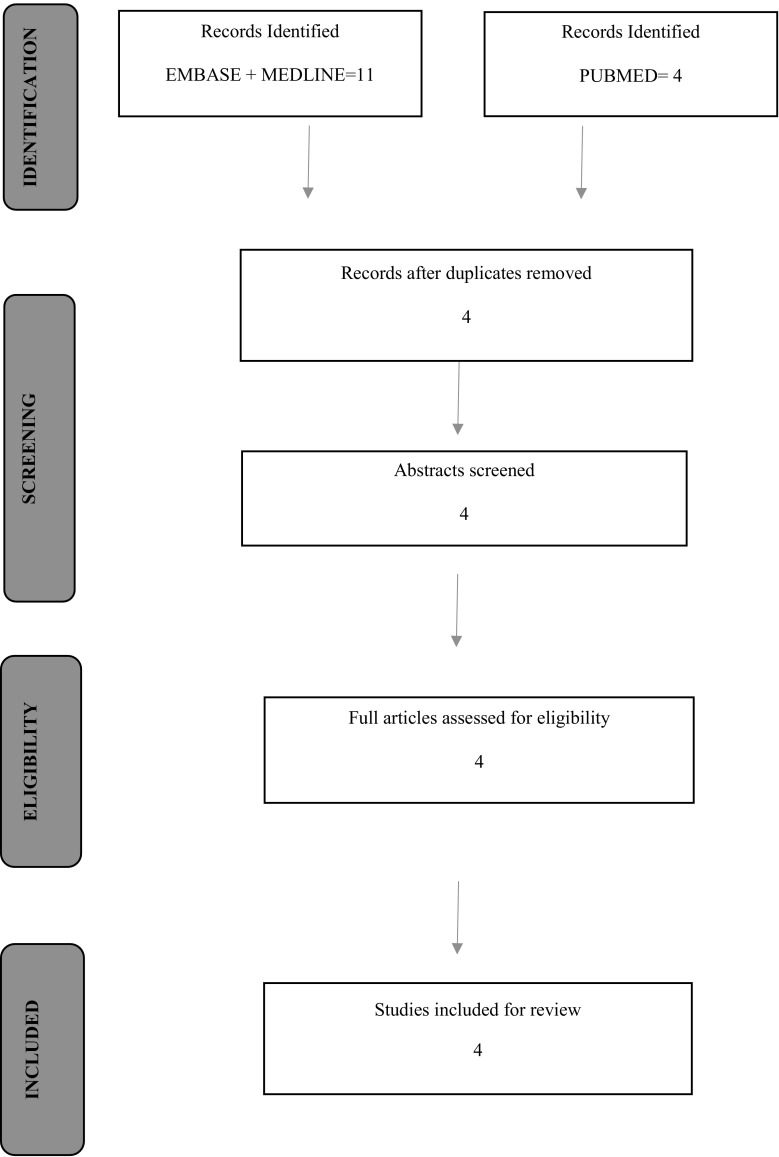
Flow chart showing search process in identifying suitable papers for analysis

Four records were identified after running searches using Embase, Medline and PubMed. The details of this process are showed on the flow chart (Fig. 1). All 4 articles were found suitable that fulfilled the eligibility criteria and were included for the review (Tables 5, 6).

**Table 5:** Summary-1

Authors	Study design	Age	Sex M: F	Previous treatment	Intervention	Pt had surgery	Final analysis	Level of study
Chan et al. [21]	Case series retrospective	37.2 (24–58)	26:4	Yes	HVIGI 10 mL 0.5% bupivacaine 25 mg hydrocortisone 4 × 10 ml of normal saline	0	21	IV
Humphrey et al. [22]	Case series prospective	43.5 ± 11.6	7:4	Yes	HVIGI 10 mL 0.5% bupivacaine 25 mg hydrocortisone 4 × 10 mL normal saline	0	11	IV
Maffulli et al. [20]	Case series prospective	37.5 (2263)	69:25	Yes	HVIGI 10 ml 0.25% bupivacaine 62,500 U aprotinin	8	87	IV
Wheeler et al. [23]	Case series retrospective two groups	50.6 ± 11.3	14:20	Yes	Group 1: 10 ml 1% lidocaine +40 ml N/saline HVIGI Group 2: dry needling + HVIGI. 10 ml 1% lidocaine +20 ml N/saline	Group 1:3 Group 2:6	34	III.

**Table 6:** Summary-2

	Outcome measures	Strengths/weakness	Conclusion
Chan et al.	Short-term study-specific questionnaire Long-term VISA-A score	*Strength* Use of validated scores Standardised management *Weakness* Selection bias Postal questionnaire Recall bias No control group	HVIGI reduce pain and improve short- and long-term functions
Humphrey et al.	VISA-A score Neovascularisation grade	*Strengths* Prospective Use of validated scores Standardised management *Weakness* Selection bias Small number No randomisation No control group	HVIGI—an effective treatment to improve symptoms, reduce neovascularisation and decrease maximal tendon thickness
Maffulli et al.	Difference in VISA-A score Neovascularisation grade	*Strengths* Prospective Approval of local ethics committee Single radiologist—board certified Independent assessor Standardised management *Weakness* Selection bias No control group	HVIGI effective in reducing pain and improve short- and long-term functions in 68% of the patients
Wheeler et al. [23]	Difference in VISA-A score	*Strengths* Compared two groups Use of validated scores *Weakness* Duration of symptoms in Group B not available Post-injection management not standardised	HVIGI reduces VISA-A score in both groups Symptoms improve more in group 1 (HVIGI using more volume without dry needling)

### Study types

Three articles selected were case series studies [20–22]. Studies were done in a single centre in London hospital. Wheeler et al. [23] compared effect of HVIGI with or without dry needling in two groups.

### Population

In the first three studies, the patients were recruited from the same centre, were physically active individuals and were private patients. The sample population in these three papers does not represent the whole population as the general population seeking treatment from NHS hospitals is a mixture of individuals with varying physical activities. The fourth study was carried out in the NHS setting, and therefore, population resemble more closely to the general population.

### Intervention

Maffulli et al. used aprotinin rather than hydrocortisone in the HVIGI. Currently, there are no trials comparing HVIGI with hydrocortisone and HVIGI with aprotinin. At present, there are some concerns with the use of aprotinin such as post-operative thrombosis, organ dysfunction and allergic reactions. Wheeler et al. used HVIGI with or without dry needling. They also did not use corticosteroids.

### Comparison

There were no control groups in the first three papers and, therefore, there was no comparison. Wheeler et al. compared the effect of HVIGI with and without dry needling.

### Outcome measures

VAS is easy to use and understand. It seems to assess more closely what patients experience. It is responsive, reliable and valid [24]. The results are recorded in normalised numeric form and, therefore, easy to compare with different patients. It can be sometimes difficult to understand by the users and require time and commitment to explain to them. It has been suggested that mark on the VAS has no interpretable meaning [24]. Chan et al. used the study-specific questionnaire, and VAS was part of it.

VISA-A score is a reliable and validated score. It provides utility in both clinical setting and research. The questionnaire can be self-administered, with minimal investigator assistance, and hence, it avoids the observer bias [25]. The study population in these three studies were all athletes but were in different sports. It is suggested that VISA-A score can be used in intervention studies, but the results would be more specific if used in homogenous group of athletes, such as runners only or volleyball players only [26].

## Results

The summary of the results is shown below (Table 7).

**Table 7:** Results summary

	VAS score	VISA-A score	USG findings
Chan et al.	*Two weeks*	At 30 weeks	×
	Pre: 76 mm	Pre: 44.8	
	Post: 25 mm	Post: 76.2	
	*At 30* *weeks*		
	Pre: 76 mm		
	Post: 28 mm		
Humphrey et al.	×	Pre: 46.3	*Thickness*
		Post: 84.1	Baseline: 8.7 mm
			3 weeks: 7.6 mm
Maffulli et al.	×	Baseline: 41.7	Thickness
		12 months: 74.6	Baseline: 9.1 mm
			12 months: 7.3 mm
Wheeler et al.	×	*Group A*	
		Pre: 30 ± 21	
		Post: 64 ± 28	
		*Group B*	
		Pre: 31 ± 14	
		Post: 37 ± 20	

### Strengths and weaknesses of the studies

The first three studies were carried out in the same centre, which was a private hospital in London. There is consistency in the papers with similar methodology, intervention and results. The authors have developed tools and protocols to consistently provide treatment for this condition. However, the normal population results cannot be compared with this group of patients. The fourth study was carried out in the general population. It was inspired by an audit. These studies can be used as baseline studies to formulate high-quality randomised controlled trials.

## Discussion

These papers have shown promising results of high-volume injection in the management of chronic Achilles tendinopathy. Wheeler et al. [23] has further added that HVIGI without dry needling is more effective than HVIGI with dry needling. Achilles tendinopathy is a difficult condition to treat. The mainstay of treatment is non-operative. Oral NSAIDs are being used, both orally and as local application, but there is no convincing evidence that they are effective [26]. Eccentric loading exercises are most commonly used with good results [19]. It can be used for 6–12 weeks. The role of shockwave therapy (SWT) is controversial, and further evidence is required to justify its use in the Achilles tendinopathy [26].

In the resistant cases, injections have been used with variable results. These include corticosteroids, sclerotherapy, autologous blood, platelet-rich plasma, high-volume injections, hyperosmolar dextrose, and aprotinin and low-dose heparin. Andres et al. [26] have described that sclerotherapy has shown promising results, but most of the papers have come from the same group of authors. However, the same can be said about the ultrasound-guided high-volume image-guided injections, as all the three papers are from the same hospital. The results of platelet-rich plasma do not show expected benefits [27]. A randomised double-blind placebo-controlled study evaluating eccentric exercises and PRP or saline injection showed no difference in improvement in pain and activity at six months. A recent meta-analysis concluded that there was no evidence of any benefit in using PRP in the treatment of Achilles tendinopathy. It may be useful in Achilles tendon repair [19].

Metcalfe et al. [28] in their systematic review have concluded that there is no consensus as to whether local glucocorticoid injections have a therapeutic role in the treatment of Achilles tendinopathy. There is a small chance of tendon damage, and therefore, risks may outweigh the potential benefits. Conversely, Chan et al. and Humphrey et al. used hydrocortisone in their injection, but there was no reported tendon rupture in their study. Wheeler et al. did not use steroids in their technique to avoid the risk of tendon damage [23].

Reviewing these four papers one can say that HVIGI have shown good early results. These injections also reduced neovascularisation in the tendon as shown by Maffulli et al. [20], but it is worth remembering that pain is also possible in the presence of normal imaging [5]. Surgery should remain as last resort and when most of the non-operative methods have failed, and the patient fully understands the risks and benefits of the surgical procedure. There is 60–80% chance of satisfactory results after the surgical procedures [26], and these results are pretty similar to HVIGI as Maffulli et al. [20] showed improvement in pain and function in 68% of the patients.

## Conclusion

We, therefore, conclude from the literature review that ultrasound-guided dry needling with high-volume injection provides good short- to medium-term relief of symptoms in the management of chronic mid-substance Achilles tendinopathy. The results are better without dry needling. It can be considered as one of the many options available for the management of this condition. It works well in conjunction with non-operative treatments, especially with eccentric loading exercises. It has low complication rate. However, based on this it is difficult to comment if this is superior to other non-operative options. Even in these four studies, there were so many variables, such as volume and the content of the solution used to achieve desired effects. Therefore, good-quality randomised controlled trials are required to find the best evidence in the management of this condition. But setting up a RCT in this case would not be easy, as there are so many options available. It is not simply comparing two treatment methods. Surgery should remain as the last resort when all the non-operative methods have failed.
